# Matrix metalloproteinase 9 and dipeptidyl peptidase-4/cluster of differentiation 26 are better performing biomarkers than matrix metalloproteinase 3 in stratifying mild and moderate activity of the ulcerative colitis

**DOI:** 10.11613/BM.2026.030702

**Published:** 2026-08-10

**Authors:** Patricija Banković Radovanović, Dijana Detel, Jadranka Cetina Žgrablić, Helena Čičak, Gabrijela Stanić, Gordana Juričić, Loredana Labinac, Manuela Perković, Rosana Troskot Perić

**Affiliations:** 1 Department of transfusion medicine, General hospital Pula, Pula, Croatia; 2 Department of medical chemistry, biochemistry and clinical chemistry, Faculty of Medicine, Rijeka, Croatia; 3 Department of gastroenterology, General hospital Pula, Pula, Croatia; 4 Department of medical laboratory diagnostics, University hospital “Sveti Duh”, Zagreb, Croatia; 5 Department of pathology and cytology, University hospital “Sveti Duh”, Zagreb, Croatia; 6 Department of medical laboratory diagnostics, General hospital Pula, Pula, Croatia; 7 Department of pathology and cytology, General hospital Pula, Pula, Croatia; 8 Department of gastroenterology and hepatology, University hospital “Sveti Duh”, Zagreb, Croatia; 9 Faculty of health studies, University of Rijeka, Rijeka, Croatia

**Keywords:** biomarkers, ulcerative colitis, ROC curve, disease progression

## Abstract

**Introduction:**

Ulcerative colitis (UC) is an idiopathic, chronic inflammatory bowel disease with an unpredictable clinical course. Reliable biomarkers reflecting intestinal inflammation remain a clinical priority. The aim is to evaluate the matrix metalloproteinase’s (MMP) 3, MMP9 and peptidase-4/cluster of differentiation 26 (DPP4/CD26) discriminative ability between different stages of the UC and assess their association with disease activity.

**Material and methods:**

This cross-sectional study enrolled 40 patients with mild and moderate UC. Disease activity was assessed using Truelove-Witts, Ulcerative Colitis Endoscopic Index of Severity (UCEIS), and Mayo score, as well as histological evaluation. Biomarker concentrations were measured in serum samples. Discriminative ability was evaluated using receiver operating characteristic (ROC) analysis with area under the curve (AUC) and Youden-derived cut-off values. The association between biomarker concentrations and disease activity was assessed using Spearman’s rank correlation coefficient.

**Results:**

MMP9 and DPP4/CD26 demonstrated comparable discriminative ability (AUC = 0.76, 95% confidence interval (CI): 0.60 to 0.88, P < 0.001; Cohen’s d = 1.09 and AUC = 0.71, 95%CI: 0.54 to 0.84, P = 0.015; Cohen’s d = 0.86, respectively). At the Youden-derived cut-off, MMP9 showed high sensitivity (80%) and moderate specificity (60%) to discriminate, whereas DPP4/CD26 showed lower sensitivity (45%) and high specificity (90%). MMP9 demonstrated statistically significant weak-to-moderate correlations with all disease activity tests (ρ = 0.41-0.49; P = 0.001-0.008), while MMP3 and DPP4/CD26 showed limited or inconsistent correlations.

**Conclusion:**

MMP9 and DPP4/CD26 displayed a significant difference between mild and moderate UC activity and demonstrated comparable discriminatory potential, although their combination does not enhance this feature. MMP9 showed a weak but significant association with disease activity, whereas MMP3 showed no clinical relevance.

## Graphical abstract

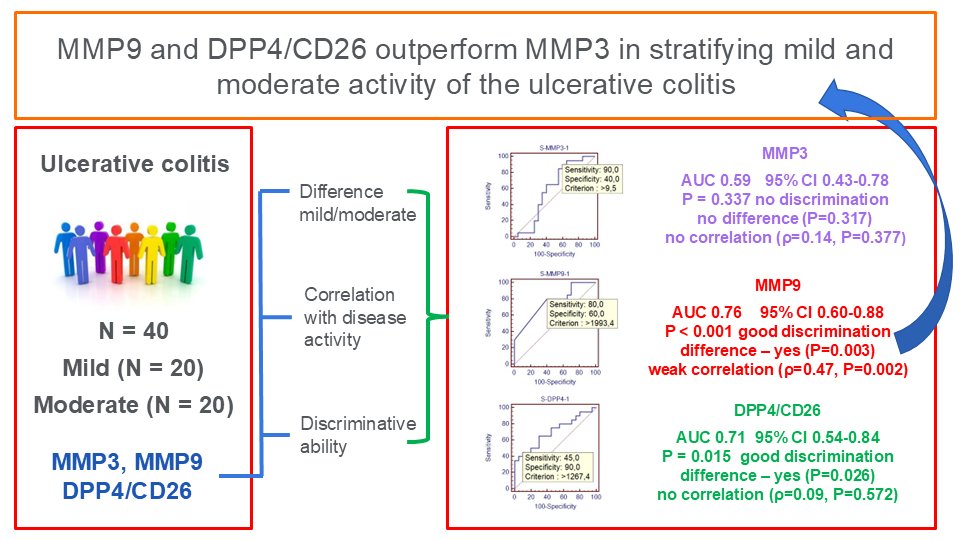

## Highlights

• Matrix metalloproteinase (MMP) 9 and dipeptidyl peptidase-4/cluster of differentiation 26 (DPP4/CD26) showed better discrimination of the mild and moderate ulcerative colitis than MMP3

• MMP9 had the highest area under the curve and largest effect size, but DPP4/CD26 demonstrated comparable discriminatory ability

• DPP4/CD26 and MMP3 demonstrated no correlations with disease severity, MMP9 showed weak correlation

• Disease severity was only partially explained by biomarkers

## Introduction

Ulcerative colitis (UC) is an idiopathic, chronic inflammatory bowel disease with an unpredictable course. Its diagnosis is primarily based on invasive diagnostic procedures, including endoscopic examination and mucosal biopsy, as well as histopathological tissue analysis along with clinical assessment of laboratory indicators (*1*). Although the etiology of this disease has not yet been fully understood, it is believed to be driven by an inappropriate immune response of the intestinal mucosa to dietary antigens and/or commensal microbiota, induced by a complex interaction of genetic changes and various environmental factors, such as stress. This is followed by an inflammatory reaction and increased production of reactive oxygen and nitrogen species, leading to tissue injury and impairment of intestinal function (*2*).

Ulcerative colitis has intrigued the scientific and professional public for decades with respect to its etiopathogenesis, diagnostics and disease monitoring as well as therapeutic strategies. Since periods of remission and relapse alternate abruptly and unpredictably during the disease, it is essential to find reliable predictors of disease progression. Such predictors would also facilitate therapy-related decisions contributing to its more rational application, as well as reducing the potential harmful effects of the administered drugs (*3*). The goal of treatment is to achieve and maintain remission with complete mucosal healing, to eliminate inflammation and improve immune status (*4*). Although the algorithm for determining therapy is well established, treatment is generally applied individually and depends on the extent and location of the inflammatory process, disease activity, clinical course of the disease and patient response to the applied therapy (*4*). Therefore, to establish a good therapeutic regimen, apart from a differential diagnosis, it is very important to be able to predict the course of the disease and the risk of relapse, preferably by a non-invasive method or procedure (*5*). In this regard, circulating biomarkers represent promising tools. The role of a range of circulating markers in serum, urine and the intestinal mucosa has been investigated to date.

Previous research has indicated a connection between C-reactive protein (CRP) and therapy outcomes as well as disease activity, although there are also studies in which CRP has proven to be an unreliable marker of activity (*6*, *7*). However, CRP as a marker of inflammation lacks disease specificity, as it is elevated in a broad spectrum of pathological conditions and is not specific to UC. Nevertheless, it still plays an important role in clinical management of many inflammatory diseases (*8*).

Furthermore, several prospective studies have investigated the prognostic potential of fecal calprotectin (fCal) in a sample of patients with UC in clinical remission, showing that a twofold increase in fCal concentrations over 3 months increases the risk of relapse and that low fCal concentrations are associated with long-term remission and mucosal healing (*9*, *10*). It has also been observed that fCal correlates with the clinical status of UC and endoscopic findings, but it can also predict remission in the patient with refractive UC (*11*, *12*).

Among other potential biomarkers, which are not routinely examined, are a group of proteolytic enzymes with diagnostic potentials. Matrix metalloproteinase (MMP) and peptidase-4/cluster of differentiation 26 (DPP4/CD26) are part of this large family of proteolytic enzymes, initially shown to play an important role in the remodelling of the extracellular matrix (*13*, *14*). Experimental models of inflammatory bowel disease (IBD) have demonstrated that these proteolytic enzymes participate in the development of various pathological conditions, especially those underpinned by inflammatory and/or malignant processes (*15*, *16*). Within MMP family, MMP3 and MMP9 have been extensively investigated as potential predictive and prognostic biomarkers of IBD (*17*-*19*). It has also been shown that MMP3 plays a critical role in the formation of fibrosis and fistulas. Therefore, the level of expression of this protease in inflamed tissue could indicate the further advancement of the disease and the risk of developing complications (*16*). Additionally, experimental evidence indicates that inhibition of metalloproteinase impair wound healing, further underscoring the crucial role of MMPs in tissue remodelling and damage (*20*).

Over the past two decades, studies have shown that DPP4/CD26, through its proteolytic effects on a wide range of substrates, is involved in the modulation and thus regulation of the immune response in UC (*14*). Studies in patients with UC have suggested that DPP4/CD26 may serve as a potential biomarker for monitoring disease activity and inflammatory burden (*21*, *22*).

However, despite promising findings, only a limited number of studies have directly linked circulating concentrations of these proteolytic enzymes with degree of inflammatory activity within intestinal tissue.

Accordingly, we hypothesized that serum concentrations of MMP3, MMP9 and DPP4/CD26 reflect ongoing inflammatory process in the bowel tissue of patients with UC. Therefore, the primary aim of the study is to explore whether the concentration of these biomarkers in blood differs in distinct stages of the UC activity (mild, moderate and severe). The second aim is to determine whether the concentration of these biomarkers in blood is correlated with different stages of the disease. The third aim is to identify which of these biomarkers show the strongest correlation with the disease stage or has better discriminative ability.

## Material and methods

### Subjects

This cross-sectional study was conducted as part of a larger prospective study, in two centres: General Hospital Pula and University Hospital “Sveti Duh” Zagreb, Croatia, between January 2020 and August 2025, with a gap of three years during the COVID-19 pandemic, when sampling was suspended and actually started in May 2023. Ethical committees of both institutions approved the study (1224/18-2 and 01-949/16) in accordance with the Helsinki Declaration.

Participants were selected by a gastroenterologist according to the established inclusion criteria after setting the diagnosis of UC based on clinical examination, medical history, endoscopic evaluation, and histological analysis of colonic biopsies. In the process of inclusion, the gastroenterologist informed the participants about the purpose of the study, study procedures, their role and rights, as well as any information that they considered relevant. Only fully informed participants who provided written informed consent were included. The aforementioned inclusion criteria were confirmed diagnosis of UC, age above 18 years, and signed informed consent. Persons with Crohn’s disease, irritable bowel syndrome, intestinal infections or indeterminate colitis, as well as pregnant women, were not included in the study.

Based on the collected data and disease activity, participants were stratified according to clinical and endoscopic classification into three groups: i) 20 participants with mild activity, ii) 20 participants with moderate activity and iii) 20 participants with severe activity.

Blood samples for biochemical analyses were collected to assess general and nutritional status (complete blood count, iron, ferritin and albumin) and inflammatory factors (white blood cell (WBC), erythrocyte sedimentation rate (ESR), CRP, ferritin) of the subjects. Three mL of K3EDTA venous blood, 8 mL of venous blood with Z Serum Separator Clot Activator tube and 1.5 mL of citrated (4NC ESR sodium citrate 3.2% tube) venous blood were collected. All the tubes used in the study were Vacuette, Greiner Bio-One Kremsmuenster, Austria. On the day of the blood collection, patients also provided a stool specimen in a sterile container for the determination of the fCal concentration.

### Methods

For the clinical assessment of the disease severity, Truelove-Witts index was used which classifies UC into three categories: mild, moderate and severe activity, based on the number of bloody stools *per* day, pulse, body temperature, hemoglobin concentration and ESR (*23*). Classification criteria are described in Supplementary table 1.

Ulcerative colitis endoscopic index for severity (UCEIS) was used for the endoscopic assessment of the disease activity (*23*). Ulcerative colitis endoscopic index for severity evaluates vascular patterns, bleeding, erosions and ulcers, with a total score ranging from 0 to 8, and it is described in Supplementary table 2. A score of 0-1 is considered a remission, 2-4 a mild activity, 5-6 a moderate and 7-8 a severe activity (*24*).

Mayo endoscopic subscore (MES) was also used for endoscopic assessment of the disease activity (*23*). With MES, gastroenterologists described the mucosal appearance at endoscopy as normal (score 0), mild activity (score 1), moderate activity (score 2) and severe activity (score 3). Classification details are presented in Supplementary table 3.

Since the original scoring systems differed in their numerical ranges, prior to analysis all scores were recoded into a unified ordinal classification based on clinically defined disease activity categories: 0 = no disease activity, 1 = mild activity, 2 = moderate activity, and 3 = severe activity. This harmonization was performed using clinically established interpretative thresholds for each scoring system (presented in Supplementary material). Following this recoding procedure, the analysed variable represented an ordinal outcome with four ordered categories consistently shared across all methods.

Biopsy samples obtained during colonoscopy were fixed in 10% buffered formalin. The collected and fixed samples were embedded in paraffin blocks and sectioned at 3.5 μm. The resulting slides were stained with hematoxylin and eosin (Merck, Sigma-Aldrich, Darmstadt, Germany) using the GEMINI AS automated stainer (Thermo Fisher Scientific, Waltham, USA). Histological analysis was performed on the colon tissue biopsies, by establishing: i) the presence of chronic inflammatory infiltrate defined by lymphocytes, plasmocytes, or eosinophils in the lamina propria; ii) the presence of acute inflammatory cell infiltrate defined by neutrophils in the lamina propria, in epithelium or the destruction of crypt by neutrophils; iii) the presence of ulceration. Findings were expressed as the Nancy histological index that is presented in Supplementary figure 1 (*25*).

Parameters of inflammation, general and nutritional status were determined from blood by using photometry (albumin) and immunoturbidimetry (CRP, iron) on the Roche Cobas c503 analyzer (Roche Diagnostics, Meinheim, Germany), CMIA (ferritin) on the Abbott Allinity I (Abbott Laboratories, Abbott Park, USA). Erythrocyte sedimentation rate was determined with the modified Westergren method on the Sed Rate Screener 100/II (SRS 100/II) (Greiner Bio-One GmBH, Kremsmünster, Austria) and complete blood count with optical flow cytometry and cytochemical method on the Sysmex XN-1000 analyzer (Sysmex Corporation, Kobe, Japan). To obtain serum samples, tubes were centrifuged for 10 min at 1800xg. For determining the concentration of MMP3, MMP9 and DPP4/CD26, serum aliquots were separated and stored at - 80°C until analysis. Serum concentrations of MMP3, MMP9, and DPP4/CD26 were determined using sandwich enzyme-linked immunosorbent assay (ELISA) kits (Proteintech, Manchester, UK). The assays were performed according to the manufacturer’s instructions using capture antibody coated on the wells, conjugate (detection antibody labelled with biotin-streptavidin-horseradish peroxidase), and tetramethylbenzidine substrate. Analytical specification for MMP3, MMP9 and DPP4/CD26 ELISA tests are provided in Supplementary table 4. Each sample was run in duplicate. A microplate reader (Bio-Tek EL808, Winooski, USA) measured the absorbance of the final product at 450 nm with the correction wavelength set at 630 nm. The concentration of MMP3, MMP9 and DPP4/CD26 in each sample was calculated from the four-parameter logistic standard curve and expressed in nanograms *per* millilitre (ng/mL).

Concentrations of fecal calprotectin were determined on the BIO-FLASH instrument (Inova Diagnostics, San Diego, USA) with QUANTA Flash Chemiluminescence Immunoassay (CLIA) and Buhlmann fCal turbo (Buhlmann Laboratories AG, Shonenbuch, Switzerland) following fecal extraction under the procedure described by the manufacturer.

Because two different fCal assay methods used in the same study could introduce method-related variability on results, we searched for similar cases. Published comparative data indicate that systematic bias between methods can be observed; in fact, constant and proportional differences between these two methods have been reported, with the following equation of regression line (*26*):

y = - 19.73 (- 37.26 to - 7.25) + 2.21 (2.02 to 2.55) x.

However, despite these discrepancies at the absolute quantitative level, agreement in clinically relevant interpretative categories remains high, with a reported kappa coefficient of 0.885 (95% confidence interval (CI): 0.76 to 1.00), indicating excellent concordance.

### Statistical analysis

The sample size was determined based on a statistical power analysis with α = 0.05 and β = 0.20. Data from previous studies on MMP3 and MMP9 as well as DPP4/CD26 in patient samples with UC were used for the power analysis. In the MedCalc spreadsheet calculator for the sample size data for each parameter were entered, specifically: the difference in the values of the observed parameter during the active phase of the disease and remission as well as the standard deviations for these data. The analysis indicated that a minimum of 19 participants *per* group was required.

To assess the normality of distribution, Kolmogorov-Smirnov test for all variables was used. However, due to the sample size bellow 30, non-parametric tests were used and data were presented as median (interquartile range). Age was presented as median and range. Differences between groups were analyzed using the Mann-Whitney U test.

With Friedman ANOVA, differences between ordinal measurements (clinical, endoscopic and histological results) were evaluated, which are presented as median and interquartile range (IQR). Associations between biomarker concentrations and disease severity scores were assessed using Spearman rank correlation test.

The discriminative ability of MMP3, MMP9 and DPP4/CD26 as well as fCal was evaluated using receiver operating characteristic (ROC) curve analysis. The optimal cut-off value for discrimination between disease stages was determined using the Youden index. Areas under the curve (AUC) were compared using DeLong test. Effect size was calculated using Cohen’s d.

We defined the level of significance as a P value less than 0.05. We performed the statistical analyses by using MedCalc 12.5.0.0 statistical software (MedCalc Software, Ostend, Belgium).

## Results

A total of 40 patients were included in the final analysis and were stratified into two groups according to disease activity: 20 patients with mild UC and 20 patients with moderate UC. Although a third group with severe disease activity was initially planned, only six patients met the inclusion criteria for this category; therefore, this group was excluded from further analysis due to insufficient sample size for reliable statistical evaluation.

The demographic data of two groups of subjects as well as laboratory results along with the assessment of the disease activity are presented in Table 1.

**Table 1:** Summary statistics of demographic data, clinical and laboratory results in patients with UC

	UC activity*		
	**Mild** **(N = 20)**	**Moderate** **(N = 20)**	**P-value^†^**
Age (years)	55 (24-85)	47 (19-76)	0.561
BMI (kg/m^2^)	25.3 (23.5-28.6)	25.7 (23.8-26.5)	0.725
WBC (x10^9^/L)	6.70 (5.52-7.75)	7.55 (6.11-8.80)	0.181
Hb (g/L)	149 (135-153)	136 (122-152)	0.168
ESR (mm/3.6 ks)	8 (5-15)	11 (7-27)	0.207
CRP (mg/L)	2.8 (0.9-5.3)	5.2 (2.2-11.1)	0.053
Ferritin (µg/L)	87.0 (52.3-169)	89.7 (26.7-134.9)	0.448
Iron (µmol/L)	12.4 (9.0-20.3)	11.6 (7.2-14.4)	0.169
Albumin (g/L)	45.0 (43.5-46.0)	45.5 (40.8-47.0)	0.914
fCal (mg/kg)	79 (36-201)	727 (275-1500)	< 0.001
MMP3 (ng/mL)	16.7 (6.1-98.4)	23.5 (14.3-55.0)	0.317
MMP9 (ng/mL)	1820.0 (733.1-2038.2)	2038.2 (2038.2-3231.1)	0.003
DPP4/CD26 (ng/ml)	798.5 (598.7-1078.2)	1051.4 (808.3-1768.5)	0.026
*The activity was determined according to the clinical and endoscopic classifications, confirmed with histopathological Nancy index (25). ^†^Mann-Whitney for independent samples. UC - ulcerative colitis. BMI - body mass index. WBC - white blood cells. Hb - hemoglobin. ESR - erythrocyte sedimentation rate. CRP - C-reactive protein. fCal - fecal calprotectin. MMP - matrix metalloproteinase. DPP4/CD26 - dipeptidyl peptidase-4/cluster of differentiation 26. P < 0.05 was considered statistically significant.			

Friedman ANOVA did not show a statistically significant difference among the four paired disease classification (P = 0.136), indicating good concordance between these methods in defining UC activity in this cohort (Table 2).

**Table 2:** Results of comparison of clinical and endoscopic scores with histological findings to assess the ability of the scores and their fitness in defining the activity of the ulcerative colitis

**Classification tools** **(median (IQR))**	**P-value** **(Friedman ANOVA)**
**Clinical scores**	0.136
Truelove-Witts 1.5 (1.0-2.0)	
**Endoscopic scores**	
UCEIS 1.0 (0-2.0)	
MES 1.0 (0-2.0)	
**Histological findings** 2.0 (1.0-2.0)	
IQR - interquartile range. UCEIS - Ulcerative colitis endoscopic index of severity. MES - Mayo endoscopic sub score. P < 0.05 was considered statistically significant.	

Correlation analysis between serum biomarker concentrations and disease activity indices is presented in Table 3. None of the biomarkers showed strong linear correlation with disease activity, even though significant weak to moderate correlations were observed for some of them. Furthermore, ROC analysis revealed that MMP9 and DPP4/CD26 discriminated between mild and moderate disease with an AUC of 0.76 (95% CI: 0.60 to 0.88) and 0.71 (95% CI: 0.54 to 0.84), respectively (Table 4). DeLong pairwise comparison of ROC curves MMP9-DPP4/CD26 showed ΔAUC 0.06 (95% CI: - 0.16 to 0.27), P = 0.599. For MMP9, the Youden index identified an optimal cut-off value of > 1993.4 yielding a sensitivity of 80% and specificity of 60% for discrimination between mild and moderate disease. For DPP4/CD26, the optimal cut-off was > 12667.4 (sensitivity 45%, specificity 90%). Large effect size was shown for both MMP9 and DPP4/CD26 (Cohen’s d = 1.09 and 0.86, respectively). Comparable results for fCal in terms of correlation with the disease activity are presented in Table 4. DeLong pairwise comparison of ROC curves fCal-MMP9 and fCal-DPP4/CD26 showed ΔAUC 0.07 0.07 (95% CI: - 0.14 to 0.27), P = 0.520 and 0.13 (95% CI: - 0.10 to 0.35), P = 0.271, respectively.

**Table 3:** Correlation of MMP3, MMP9 and DPP4/CD26 with disease severity in patients with ulcerative colitis

	**Truelove-Witts** **Sp ρ** **(P-value)**	**UCEIS** **Sp ρ** **(P-value)**	**Mayo** **Sp ρ** **(P-value)**	**Nancy** **Sp ρ** **(P-value)**
MMP3 (ng/mL)	0.16 (0.323)	0.14 (0.377)	0.11 (0.516)	0.42 (0.007)
MMP9 (ng/mL)	0.48 (0.002)	0.47 (0.002)	0.41 (0.008)	0.49 (0.001)
DPP4/CD26 (ng/mL)	0.36 (0.025)	0.09 (0.572)	0.02 (0.893)	0.14 (0.404)
fCal (mg/kg)	0.57 (< 0.001)	0.54 (< 0.001)	0.52 (< 0.001)	0.57 (< 0.001)
Disease severity was established with clinical (Truelove-Witts), endoscopic scores (UCEIS, Mayo) and histological Nancy index (25), with addition of comparable data for fecal calprotectin. MMP - matrix metalloproteinase. DPP4/CD26 - dipeptidyl peptidase-4/cluster of differentiation 26. fCal - fecal calprotectin. UCEIS - Ulcerative colitis endoscopic Index of severity. Sp ρ - Spearman’s rank correlation coefficient. P < 0.05 was considered statistically significant.				

**Table 4:** Group comparison and discriminative ability of disease severity of MMP3, MMP9 and DPP4/CD26 in patients with ulcerative colitis (with addition of the comparable data for fecal calprotectin)

	**UC** **mild** **(N = 20)**	Activity* **moderate** **(N = 20)**	**P-value^†^**	**AUC** **(95% CI)** **P-value**	**Youden cut-off**	**Se (%)**	**Sp (%)**	**Cohen’s d^‡^**	**Interpretation** **of cut-off**
MMP 3 (ng/mL)	16.7 (6.1-98.4)	23.5 (14.3-55.0)	0.317	0.59 (0.43-0.78) 0.337	-	-	-	-	No discrimination
MMP9 (ng/mL)	1820.0 (733.1-2038.2)	2038.2 (2038.2-3231.1)	0.003	0.76 (0.60-0.88) < 0.001	> 1993.4	80	60	1.09	Good for detecting moderate disease
DPP4/CD26 (ng/mL)	798.5 (598.7-1078.2)	1051.4 (808.3-1768.5)	0.026	0.71 (0.54-0.84) 0.015	> 1267.4	45	90	0.86	Good for confirming mild disease
fCal (mg/kg)	79 (36-201)	727 (275-1500)	< 0.001	0.83 (0.68-0.93) < 0.0001	> 254	75	90	-	Good for confirming mild disease with added detection value
*The activity was determined according to the clinical and endoscopic classifications, confirmed with histopathological Nancy index (25). ^†^Mann-Whitney for independent samples. ^‡^Calculated on socsistatistics.com/effectsize/deault3.aspx; accessed January 8th 2026. UC - ulcerative colitis. MMP - matrix metalloproteinase. DPP4/CD26 - dipeptidyl peptidase-4/cluster of differentiation 26. fCal - fecal calprotectin. AUC - area under the curve. Se - sensitivity. Sp - specificity. P < 0.05 was considered statistically significant.									

## Discussion

In our study, MMP9 and DPP4/CD26 demonstrated comparable discriminative ability in differentiating between mild and moderate UC. This is supported by relatively large AUC obtained for both biomarkers and at the same time the absence of the statistically significant difference between their AUC values. In addition, MMP9 showed particular utility in identifying patients with moderate disease activity and in detecting progression from mild to moderate UC. Furthermore, the large effect size observed for both biomarkers further suggests their clinical relevance in distinguishing between two stages of disease activity. In contrast, MMP3 showed no discriminative ability and association with disease severity was limited to the histological score only, indicating low utility for disease stratifications.

Among the evaluated biomarkers, MMP9 showed the strongest overall performance with significant group differences, the highest AUC, large effect size and consistent correlations with disease severity across multiple ordinal tests, including the Truelove-Witts clinical score, UCEIS, Mayo endoscopic sub score, and the Nancy histological index. However, despite statistical significance, the magnitude of these correlations was only weak to moderate. The coefficients of determination indicate that less than one quarter of the variability in disease severity could be explained by serum MMP9 concentrations. This suggests that MMP9 reflects disease severity only partially and that additional clinical, immunological, and molecular factors substantially contribute to disease progression in UC.

Dipeptidyl peptidase-4/cluster of differentiation 26 also demonstrated significant concentration difference in mild and moderate UC, along with adequate discriminative ability and large effect size. However, its correlations with disease severity indices were inconsistent and limited to the clinical score only. This finding suggests that DPP4/CD26 may be useful for differentiating disease stages without reflecting gradual disease progression.

Moreover, results for sensitivity and specificity obtained at the cut-off values for both MMP9 and DPP4/CD26 suggest that they perform well at detecting moderate disease and confirming mild disease, respectively.

By observing demographic and laboratory data in mild and moderate group of the UC activity, it was noticed that the two groups were comparable in terms of age, body mass index, and most laboratory parameters, with no statistically significant differences observed for WBC, hemoglobin, ESR, ferritin, iron, albumin, or CRP. A pronounced and statistically significant difference was observed for fCal, which was markedly higher in the moderate activity group compared to the mild group, confirming its strong association with intestinal inflammatory burden and supporting the correct stratification of patients according to disease activity. In addition, fCal demonstrated moderate positive correlation with all applied measures of disease activity, including the Truelove-Witts clinical score, Mayo endoscopic sub score and the Nancy histological index. This is in concordance with the results of Ryu *et al.* who also demonstrated moderate positive correlation of fCal with endoscopic activity in UC assessed with Mayo end UCEIS endoscopic scores (*27*).

Due all these characteristics of the fCal, it would be anticipated that fCal, in combination with MMP9 or DPP4/CD26, could enhance the stratification between mild and moderate UC. However, the results of the further evaluation did not support this assumption. Although numerical differences in AUCs were observed between certain biomarker pairs, DeLong’s test did not demonstrate statistically significant improvements in discrimination. These findings suggest that the biomarkers provide largely overlapping diagnostic information, and that their combined use does not meaningfully enhance stratification between mild and moderately active disease. This may be explained by the fact that both MMP9 and DPP4/CD26 participate in closely related inflammatory pathways, thereby reflecting overlapping aspects of mucosal inflammation rather than distinct biological processes.

Individual results for the fCal ROC analysis in our study are in concordance with the results from the study of Swaminathan *et al*. Their results of AUC and 95%CI for fCal at the cut-off value of ≥ 250mg/kg, being associated with the active endoscopic disease, as well as the sensitivity and specificity at the derived cut-off are comparable to those in our study (*28*).

Even though there are relatively small number of similar studies of MMPs and DPP4/CD26 in UC to be compared with, some studies are partially consistent with our results. In their study, Siloşi *et al.* demonstrated statistically significant differences in MMP3 and MMP9 serum concentrations between moderate and mild activity in UC patients, although they did not test discriminatory ability of these biomarkers (*18*). Furthermore, Pinto-Lopes *et al.* reported that serum DPP4/CD26 concentrations were able to discriminate between active disease and remission in UC patients, supporting its potential role as a biomarker of disease activity (*21*).

The main limitations of this study include the small sample size despite the results of the power analysis, the exclusion of patients with severe disease activity as well as the exploratory nature of derived cut-off values. Nevertheless, the consistency of findings across multiple analytical approaches, including group comparisons, ROC analysis, effect size estimation, and correlation with independent disease activity indices, support the robustness of the observed differences.

The use of two different analytical methods for fCal should be mentioned as possible limitation. Although this reflects real-world clinical practice, inter-method variability cannot be entirely excluded. However, available evidence indicates high agreement in clinically relevant interpretative categories between assays, and in our dataset fCal values obtained with both methods showed consistent and significant correlations with clinical, endoscopic and histological findings. It should also be noted that fCal was not the primary focus of this study, but rather served as a complementary biomarker within a broader analytical framework. Therefore, potential inter-method variability is unlikely to have materially influenced the main study outcomes or overall conclusions. Nevertheless, the use of multiple analytical platforms may have introduced a degree of measurement variability that could not be fully controlled for and represents a potential source of bias. Future studies using a single standardized assay or direct head-to-head method comparison within the same cohort would be valuable to further validate these findings. Despite these limitations, we believe that the strong and consistent associations with independent markers of disease activity support the robustness and clinical relevance of our conclusions.

Furthermore, the cut-off values derived using the Youden index should be interpreted with caution, as they were obtained in a relatively small cohort and are intended only as exploratory mean for classification of disease severity. Validation in larger and independent patient populations is required before their potential clinical implementation.

In conclusion, significant differences in serum biomarker concentration between mild and moderate UC activity were observed for MMP9 and DPP4/CD26, whereas MMP3 did not demonstrate meaningful discriminatory potential. With respect to the association between biomarker concentrations and disease activity, no consistent correlation across disease stages was identified, except for MMP9, which demonstrated a weak but statistically significant association with the disease activity. Finally, both MMP9 and DPP4/CD26 exhibited comparable discriminative ability in distinguishing mild and moderate UC activity, supporting their potential utility in disease activity stratification. However, their combined use does not enhance this stratification. In contrast, MMP3 appears to have no clinical relevance in this context.

## Data Availability

The data will be shared on reasonable request to the corresponding author.
